# Peer Review of “A Local Community-Based Social Network for Mental Health and Well-being (Quokka): Exploratory Feasibility Study”

**DOI:** 10.2196/33923

**Published:** 2021-10-27

**Keywords:** local social network, community health, well-being, digital health, consumer health

*This is a peer-review report submitted for the paper “A Local Community-Based Social Network for Mental Health and Well-being (Quokka): Exploratory Feasibility Study.”*

## Round 1 Review

### General Comments

This paper [1] describes a study on a social network intervention to promote well-being in college students using local and community-based activities. The manuscript describes an exploratory feasibility study of the interventions, called Quokka. The authors ran a 6- to 8-week challenge across 4 universities. Three hypotheses were tested.

The paper is well written and will be of interest to JMIR readers; I enjoyed reading it myself and think there is great value in this type of study, particularly now given the many challenges to our mental health due to the COVID-19 pandemic and the move to predominantly online life. I would recommend “probably accept pending some revisions” to clarify some issues in data analysis and presentation.

### Specific Comments

The authors describe the challenges embedded in Quokka, and they present an overview of the program; however, they do not describe the features of the system. A walkthrough of the system would be appropriate to include.

I was confused by the testing of the 3 hypotheses. In the *Results* section, under *Evaluation Outcomes*, the authors first claim “All 3 hypotheses were confirmed”; then, they suggest “we reject the hypothesis that similar proportion of users would participate in local and remote activities during the challenges,” which seems to be H1. Please clarify and also refer to the hypothesis number (eg, H1) when discussing it.

I am not an expert in the approach taken for data analysis. I was hoping to see a better description of the steps taken to analyze the qualitative data to test for significance. I think this is necessary to improve the reliability of the outcomes.

Similarly, I was hoping to see a better description of how the thematic analysis was conducted. What was the approach in coding and forming themes? The themes are described, but we don't get any information about how these themes were identified.

*Limitations* section: I would suggest outlining the limitations of the method and data analysis given that it mainly relied on qualitative data. I do not mean to suggest qualitative data is not valid (quite the opposite); however, the authors have taken the approach to test hypotheses using the qualitative study. So a better engagement with the limitations of their approach would be appropriate.

Ethics: was the study approved by an ethics committee? How was consent obtained? Please specify.

## Round 2 Review

### General Comments

The revised manuscript provides the details requested previously. The walkthrough of the system that is added is helpful to understand its utility. Details on hypotheses and limitations also address initial concerns around clarity of the manuscript. I am happy with the changes made and recommend accepting the manuscript.

### Specific Comments

#### Minor Comments

There were a few errors in writing which I am sure will be easy to address once the authors proofread their manuscript; for instance, they used “patient” instead of “user” in some places.
